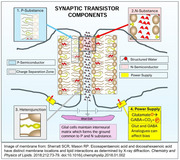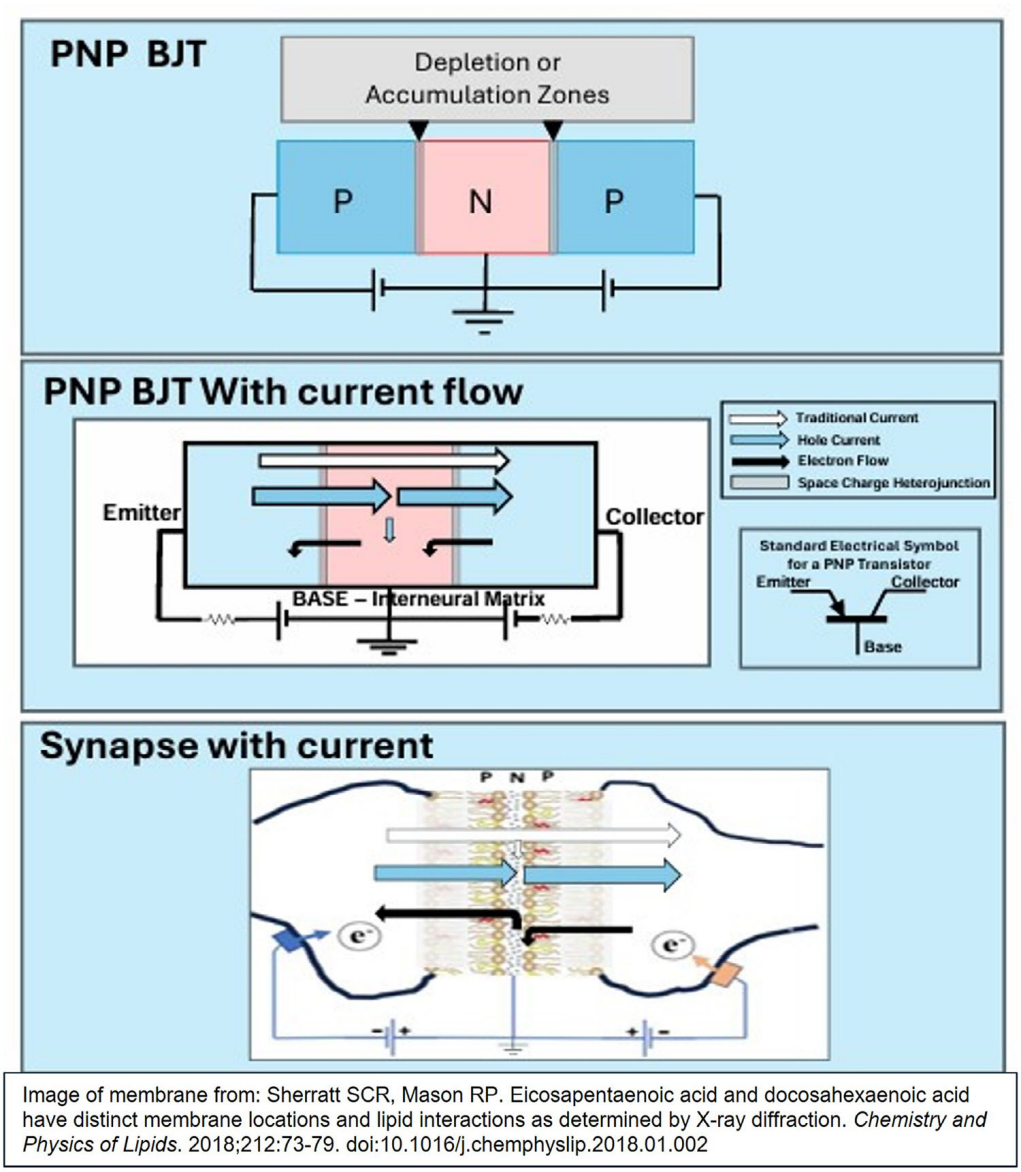# Mild Cognitive Impairment and Early Alzheimer's Diseased Explained by 21st Century Model of the Neural Synapse as a Transistor

**DOI:** 10.1002/alz70855_096383

**Published:** 2025-12-23

**Authors:** Richard C. Dobson

**Affiliations:** ^1^ Rochester Institute of Technology, Rochester, NY, USA; Osher Lifelong Learning Institute at RIT, Rochester, NY, USA

## Abstract

**Background:**

In the late 1940s, the Hodgkin and Huxley model of ion transport as the basis for neural signal transmission was born. A revolution in pharmaceutical management of many diseases ensued. Bardeen et al. developed the first transistor. The technological revolution in our lives ensued. Neural signaling as electromagnetism, prominent before then, faded. But recently the synapse has been modeled as a PNP Bipolar Junction Transistor (BJT), with the electron as the neurotransmitter, while the traditional “neurotransmitters” are vital modulators of the electrical milieu (bias as engineers say) that determines the operation of the BJT. Alzheimer's Disease (AD) is characterized by decreased signaling, usually ascribed to Tau and Aβ. A transistor model provides new insight into the atomic level changes that cause Mild Cognitive Impairment and early AD.

**Method:**

Combining concepts and knowledge of synaptic structure and composition, transistors, organic semiconductors (SCs), and quantum chemistry, a model of the synaptic transistor explains MCI and early AD.

**Result:**

A PNP‐BJT has 4 critical components: an *N*‐type SC sandwiched by 2 P SCs, an intrinsic power supply, and a “space charge” region at each *p*‐N junction. The corresponding structures in the synapse are:

1. *N*‐substance: The two lipid polar heads of the synapse with the structured water in the cleft;

2. 2 P substances: The hydrocarbon chain of the outer leaf of each synaptic membrane with docosahexaenoic acid (DHA) with its 6 pi bonds;

3. Intrinsic Power Supply: Peri‐synaptic Glutamate(Glu) receptors converting Glu(Negatively charged) to GABA(Neutral) + CO2 (Neutral) + **e‐**;

4. Space Charge Heterojunction: Molecular orbital distribution or energy levels affecting electron density at the space around DHA carbon chain and the polar head.

**Conclusion:**

All critical synaptic BJT components and their function are altered in MCI and early AD. MCI and early AD are characterized by decreased DHA (decreasing electron and hole conduction through the P SC), altered glutamate receptors (affecting the intrinsic power supply to the synaptic BJT), and hyperphosphorylated Tau affecting electrical bias mostly via changes in electrochemical properties of the interstitial matrix which forms the base terminal of the transistor. These processes are independent of tangles and plaques from late AD.